# Antibacterial and Immunomodulatory Activities of a Natural Polysaccharide Against *Staphylococcus aureus* Infection

**DOI:** 10.1002/fsn3.72411

**Published:** 2026-09-29

**Authors:** Xu‐Chen Xia, Ya‐Qi Peng, Lu‐Xuan Liu, Bing Cao, Xue‐Kun Zhong, Xi‐Dong Wu, Tao Liu

**Affiliations:** ^1^ Department of Otolaryngology‐Head and Neck Surgery, Guangdong Provincial People's Hospital (Guangdong Academy of Medical Sciences) Southern Medical University Guangzhou China; ^2^ Department of Otolaryngology‐Head and Neck Surgery, Ganzhou Hospital of Guangdong Provincial People's Hospital Ganzhou Municipal Hospital Ganzhou China; ^3^ Department of Drug Safety Evaluation Center Jiangxi Medical Device Testing Center Nanchang China; ^4^ Key Laboratory of Modern Preparations of Chinese Medicine, Ministry of Education Jiangxi University of Chinese Medicine Nanchang China

**Keywords:** antibacterial activity, immunomodulation, natural therapeutic agent, *Polygonatum kingianum polysaccharide*, rhinosinusitis, *Staphylococcus aureus*

## Abstract

Over decades, the growing prevalence of antibiotic‐resistant bacterial infections, including rhinosinusitis caused by *Staphylococcus aureus* , has represented a significant public health challenge. The emergence of multidrug‐resistant strains and biofilm formation has limited the efficacy of conventional antibiotics, leading to increased patient morbidity and an urgent need to explore alternative therapeutic agents. *Polygonatum kingianum polysaccharides* (PKP), key bioactive components derived from traditional herbs, have attracted attention for their broad pharmacological effects. However, a thorough evaluation of their antibacterial potential in complex in vivo infection models remains lacking. Herein, we report the extraction of PKP and a comprehensive assessment of its antibacterial activity both in vitro and in vivo. In vitro, PKP exhibited potent, concentration‐dependent antibacterial activity against *S. aureus* and *Escherichia coli* , with significantly stronger bactericidal activity against the Gram‐positive bacterium (89.8% at 400 mg/mL; MIC = 400 mg/mL; MBC = 700 mg/mL). The antibacterial mechanism appeared to involve disruption of the cell membrane, with structural differences among bacterial envelopes likely explaining the variation in efficacy. Furthermore, in a rat model of *S. aureus* ‐induced rhinosinusitis, intranasal administration of PKP effectively attenuated bacterial infection, as evidenced by significantly reduced neutrophilic infiltration and abscess formation, as well as decreased levels of the pro‐inflammatory cytokine interleukin‐6 (IL‐6). Meanwhile, PKP helped restore the barrier‐protective cytokine IL‐22. Hematological profiles further confirmed the systemic anti‐inflammatory and immunomodulatory effects of PKP, including regulation of leukocyte counts and improvement of immune organ indices. This study demonstrated that PKP exhibited significant in vitro and in vivo antibacterial activity against *S. aureus* through a dual mechanism of direct bactericidal action and modulation of the immune response. Overall, these findings suggest that PKP has great potential as a natural therapeutic agent for bacterial rhinosinusitis.

## Graphical abstract

Polysaccharides from *Polygonatum kingianum* (PKP) were extracted with ethanol and hot distilled water, followed by centrifugation and drying. In vitro, PKP inhibited both *E. coli* and *Staphylococcus aureus* in a concentration‐dependent manner, with stronger activity against the Gram‐positive *S. aureus* (89.8% at 400 mg/mL) than against *E. coli* (72.1% at 600 mg/mL). In a rat model of *S. aureus* ‐induced rhinosinusitis, once‐daily PKP administration for 14 days reduced neutrophilic infiltration and abscess formation, decreased the pro‐inflammatory cytokine IL‐6, and increased the barrier‐protective cytokine IL‐22. Hematological and histopathological analyses further supported systemic anti‐inflammatory and immunomodulatory effects, including regulation of leukocyte counts, reduced sinus neutrophil infiltration, and improved immune organ indices.

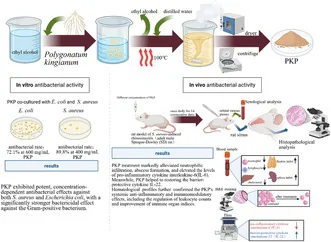

## 1 Introduction

The increasing prevalence of antimicrobial resistance (AMR) poses a severe global threat to public health, underscoring the urgent need to develop novel anti‐infective strategies beyond conventional antibiotics. The latest systematic analysis indicated that bacterial AMR was associated with approximately 4.95 million deaths in 2019, with projections suggesting that this burden will continue to rise through 2050 if left unaddressed (Naghavi et al. 2024). *Staphylococcus aureus* , particularly, is a leading pathogen responsible for a wide spectrum of infections, including the upper respiratory tract such as bacterial rhinosinusitis (Shaghayegh et al. 2022). The clinical management of rhinosinusitis is often complicated by the formation of resilient biofilms and the emergence of multidrug‐resistant strains, such as MRSA (methicillin‐resistant *S. aureus* ). These factors collectively shield bacteria from antibiotic penetration and action, compromising therapeutic efficacy and leading to chronic, recalcitrant infections (Chegini et al. 2023; Sauer et al. 2022). Indeed, *S. aureus* biofilm‐secreted factors have been shown to cause mucosal damage, mast cell infiltration, and goblet cell hyperplasia in a rat rhinosinusitis model, underscoring the direct pathogenic role of biofilms in disease progression (Houtak et al. 2024). Consequently, there is growing interest in exploring alternative or adjunctive therapeutics derived from natural products, particularly those with multi‐target effects and favorable safety profiles and a lower propensity for inducing resistance (MacNair et al. 2024).

*Polygonatum kingianum* (*PKP*), a perennial plant of the Liliaceae family widely used in Traditional Chinese Medicine, is recognized for its tonifying, antiaging, and moistening properties (Fan et al. 2025). Contemporary pharmacological studies have revealed that polysaccharides are among its primary bioactive constituents, responsible for a range of activities including immunomodulation, antioxidant, antidiabetic, and antitumor effects (Zhao et al. 2018). Among these, the immunomodulatory capacity of polysaccharides is particularly relevant for infectious diseases, as a properly regulated host immune response is crucial for pathogen clearance and tissue repair (Murphy et al. 2023). Although polysaccharides from related species, such as *Polygonatum cyrtonema Hua*, have been suggested to possess antibacterial properties (Gong et al. 2024; Lin et al. 2024), a systematic and mechanistic investigation into the direct and host‐mediated antibacterial effects of PKP is still lacking.

Furthermore, a significant gap exists between in vitro bioactivity screening and demonstrated efficacy in physiologically relevant in vivo models. The dynamic interplay between a potential antimicrobial agent and the host immune system cannot be fully captured in vitro. Many candidate agents with promising in vitro bactericidal activity fail in vivo due to an inability to modulate pathological immune responses or counteract the host‐derived inflammatory damage (MacNair et al. 2024). Therefore, a translational approach is imperative to determine whether in vitro activity translates into effective infection control in living organisms and to elucidate whether these effects are mediated by direct microbial inhibition, host immune modulation, or a synergistic combination of both.

The PKP used in this study was structurally characterized in our previous work (Fan et al. 2025). It is a mixed‐type fructan with an average molecular weight of approximately 3.0 kDa. Monosaccharide analysis revealed that PKP consists predominantly of fructose (91.3%), with smaller amounts of glucose (6.9%) and mannose (0.9%), plus trace quantities of galacturonic acid, galactose, xylose, and arabinose. NMR spectroscopy confirmed a fructan backbone comprising both β‐2,1‐ and β‐2,6‐linked fructofuranose residues, with an approximate ratio of 2.5:1, and terminal α‐glucopyranose units. Given the documented bioactivities of PKP in our previous work, we hypothesized that PKP would demonstrate effective antibacterial activity against *S. aureus* through a dual mechanism: direct bactericidal action and modulation of host inflammatory and barrier‐protective responses.

To verify this hypothesis, we designed the study with three objectives: (1) to systematically assess the in vitro concentration‐dependent antibacterial activity of purified PKP against both Gram‐positive ( *S. aureus* ) and Gram‐negative ( *Escherichia coli* ) bacteria, and to preliminarily elucidate the potential mechanism underlying the observed differential efficacy; (2) to establish a rat model of *S. aureus* ‐induced rhinosinusitis to validate the in vivo therapeutic potential of PKP; and (3) to elucidate the underlying immunomodulatory mechanisms through histopathological analysis, cytokine profiling (focusing on the pro‐inflammatory IL‐6 and the barrier‐protective IL‐22), and comprehensive hematological and immunological parameters following PKP treatment.

Together, these investigations provide a solid, clinically relevant foundation for developing PKP as a potential natural therapeutic agent for bacterial infections, with specific translational implications for managing antibiotic‐resistant rhinosinusitis.

## 2 Materials and Methods

### 2.1 Isolation and Structural Characterization of PKP

PKP was isolated from *Polygonatum kingianum* rhizomes (Yipuyuan Huangjing Technology Co. Ltd., Xinhua, China) by sequential aqueous‐ethanol defatting, hot‐water extraction, and ethanol precipitation, following the protocol described by our previous work (Fan et al. 2025). Dried roots (200 g) were processed to yield 35.92 g of PKP, with a carbohydrate purity of 99.4% as determined by the phenol–sulfuric acid assay. The molecular weight distribution, monosaccharide composition, and glycosidic linkage pattern were elucidated by HPGPC, HILIC‐CAD, PMP‐HPLC, and 2D NMR (HMBC/HMQC), respectively, as reported previously (Fan et al. 2025).

### 2.2 Antibacterial Activity

#### 2.2.1 Microbial Strains and Culture Conditions

The bacterial strains used in this study, namely *E. coli* ATCC 25922 and *S. aureus* ATCC 25923 (American Type Culture Collection, Manassas, VA, USA), were obtained from our laboratory stock culture collection. These strains are publicly available reference isolates that have been maintained in our laboratory since acquisition. A single colony of each strain was aseptically inoculated into 10 mL of sterile Tryptic Soy Broth (TSB) and incubated overnight at 37°C under static conditions to obtain active pre‐cultures.

#### 2.2.2 Preparation of Culture Media and Reagents

Nutrient Agar was prepared according to the manufacturer's instructions and sterilized by autoclaving at 121°C for 15 min. Subsequently, approximately 15–20 mL of the molten agar was poured into sterile Petri dishes and allowed to solidify at room temperature. A total of 15 plates were prepared in advance for the subsequent plating steps.

PKP was dissolved in sterile TSB to prepare a stock solution, which was then serially diluted to achieve final working concentrations of 200, 400, 600, 800, 1000, and 1200 mg/mL. All solutions were prepared fresh on the day of the experiment.

#### 2.2.3 Preparation of Bacterial Inoculum

Following overnight incubation, the bacterial cultures were adjusted to a density of approximately 2 × 10^6^ colony forming units per milliliter (CFU/mL) by preparing a 1000‐fold dilution in sterile phosphate buffered saline (PBS). The optical density at 600 nm (OD_600_) was verified against a pre‐established standard curve to confirm the target concentration.

#### 2.2.4 Assessment of Antibacterial Activity

The antibacterial activity of PKP was evaluated using a broth dilution method in a 24‐well cell culture plate. For the test groups, 200 μL of each PKP concentration (200–1200 mg/mL) was mixed with 200 μL of the prepared bacterial suspension (2 × 10^6^ CFU/mL) in respective wells, resulting in a final volume of 400 μL and final PKP concentrations of 100, 200, 300, 400, 500, and 600 mg/mL.

The experimental setup included the following controls:

- Positive control: 200 μL of bacterial suspension was mixed with 200 μL of sterile TSB (no PKP).
- Negative control: 400 μL of sterile TSB only (no bacteria, no PKP) was added to monitor for contamination.

The plate layout was designed as follows: bacterial test groups for a single strain were allocated to wells A1–A5, the corresponding positive control was placed in well A6, and the negative control was placed in well B1. This layout was replicated for the second bacterial strain. The plate was then covered and incubated at 37°C for 24 h.

#### 2.2.5 Determination of Minimum Inhibitory Concentration (MIC) and Minimum Bactericidal Concentration (MBC)

The MIC and MBC of PKP against *S. aureus* were determined using a modified broth microdilution method in 96‐well microtiter plates. Briefly, 100 μL of *S. aureus* suspension (2 × 10^6^ CFU/mL) was added to each well containing 100 μL of serially diluted PKP in sterile TSB, yielding final PKP concentrations ranging from 100 to 800 mg/mL. The plates were incubated at 37°C for 24 h. The MIC was defined as the lowest concentration of PKP that completely inhibited visible bacterial growth (no turbidity) after incubation.

To determine the MBC, 100 μL aliquots from wells showing no visible growth were spread onto nutrient agar plates and incubated at 37°C for 12 h. The MBC was defined as the lowest concentration of PKP that resulted in ≥ 99.9% killing of the initial inoculum (i.e., no colony formation or < 20 CFU/mL, corresponding to a 3‐log reduction from the initial 2 × 10^6^ CFU/mL). All experiments were performed in triplicate.

#### 2.2.6 Bacterial Enumeration and Determination of Antibacterial Rate

After the 24‐h coculture period, the bacterial suspensions from each well were subjected to serial dilution to achieve a quantifiable number of colonies. Briefly, 100 μL of the cultured suspension was transferred into 900 μL of sterile PBS, generating a 10‐fold dilution. This process was repeated serially to obtain dilution factors of 10^4^.

From each of these dilution tubes, 100 μL aliquots were spread evenly onto the pre‐prepared nutrient agar plates. The plates were incubated at 37°C for 12 h to allow for the development of distinct colonies.

The number of viable bacteria was determined by counting the colonies on plates containing 30–300 colonies. The count from the positive control group (without PKP) was designated as *N*_0_, representing 0% inhibition. The counts from the PKP‐treated groups were designated as *N*_1_.

The antibacterial rate (AR) was calculated using the following formula:

$$\text{Antibacterial rate}\;\left(\%\right)=\left[\left(N_{0}-N_{1}\right)/N_{0}\right]\times 100\%$$

All experiments were performed in triplicate to ensure reproducibility, and the data are presented as the mean ± standard deviation.

#### 2.2.7 Time‐Kill Kinetic Assay

The bactericidal or bacteriostatic nature of PKP and its rate of action against *S. aureus* were evaluated by a time‐kill kinetic assay. Briefly, an overnight culture of *S. aureus* was adjusted to a density of 1.0 × 10^8^ CFU/mL in Mueller‐Hinton (MH) broth. Aliquots (200 μL) of the bacterial suspension were dispensed into each well of a sterile 96‐well flat‐bottom microtiter plate. PKP was added to final concentrations of 1 × MIC (400 mg/mL) and 2 × MIC (800 mg/mL). Control wells received 200 μL of bacterial suspension without PKP. The plate was incubated at 37°C with shaking at 150 rpm in a microplate reader chamber.

The OD_600_ was measured every 4 h over a 24‐h period (0, 4, 8, 12, 16, 20, and 24 h) using a microplate reader. Prior to each reading, the plate was briefly shaken for 30 s to ensure uniform bacterial dispersion. The OD_600_ values were blank‐corrected against sterile MH broth. Bacterial growth curves were generated by plotting OD_600_ versus time. All assays were performed in triplicate in three independent experiments.

#### 2.2.8 Biofilm Eradication Assay

Bacterial biofilms are three‐dimensional structures formed by bacteria to adapt to the environment, mainly composed of bacteria and their secreted extracellular polymers. Therefore, this study evaluated the scavenging effect of the material on the mature biofilm of bacteria. Briefly, an overnight culture of *S. aureus* was adjusted to a density of 1.0 × 10^8^ CFU/mL in Mueller‐Hinton (MH) broth. Aliquots (1 mL) of the bacterial suspension were dispensed into each well of a 24‐well flat‐bottom microtiter plate. The plate was sealed with Parafilm and incubated at room temperature in the dark for 4 h to allow bacterial sedimentation and initial adhesion to the well surface. Subsequently, the plate was transferred to a 37°C incubator and incubated statically for 36 h to establish mature biofilms.

Following biofilm maturation, 500 μL of the supernatant was carefully aspirated from each well to remove planktonic and loosely adherent bacteria. The biofilms were then challenged with PKP at concentrations of 1/2 MIC (200 mg/mL), MIC (400 mg/mL), and 2 × MIC (800 mg/mL). Specifically, 500 μL of fresh MH broth containing the respective concentration of PKP was gently added to each well every 24 h for two consecutive days. Control wells received 500 μL of fresh MH broth without PKP. The plate was incubated statically at 37°C throughout the treatment period.

After the final treatment, the culture medium was completely aspirated from all wells. The biofilms were fixed with 1 mL of 2.5% glutaraldehyde solution at room temperature for 15–30 min. The fixative was then removed, and the wells were gently rinsed twice with sterile PBS. The adhered biofilms were stained with 500 μL of 0.1% (w/v) crystal violet aqueous solution at room temperature for 10–30 min. Excess dye was removed by aspirating the staining solution and washing the wells three times with distilled water. The crystal violet bound to the biofilm biomass was solubilized by adding 200 μL of 33% (v/v) glacial acetic acid to each well, followed by incubation at room temperature with gentle shaking for 10–15 min. The resulting solutions were transferred to a new 96‐well plate (100–200 μL per well), and the absorbance was measured at 570 nm using a microplate reader. The biofilm biomass was expressed as a percentage relative to the untreated control:

$$\text{Biofilm biomass}\;\left(\%\right)=\left({\mathrm{OD}}_{570}\;\text{of treated well}/{\mathrm{OD}}_{570}\;\text{of control well}\right)\times 100\%.$$

All assays were performed in triplicate in three independent experiments.

### 2.3 Antibacterial Activity and Eradication of *S. aureus* Infection In Vivo

#### 2.3.1 Animals and Ethical Statement

Sixty adult male Sprague–Dawley (SD) rats (weighing 200–250 g) were housed under standard laboratory conditions (room temperature: 22°C–28°C; 12‐h light/dark cycle) with free access to a standard diet and sterile water. After a 2‐week acclimatization period, during which all animals exhibited normal feeding, drinking, behavior, and mental status, the experiments commenced. All animal procedures were conducted in accordance with the National Institutes of Health Guide for the care and use of laboratory animals and were approved by the Institutional Animal Care and Use Committee (IACUC) of the Department of Drug Safety Evaluation, Jiangxi Testing Center of Medical Instruments, Nanchang, China (approval no. JXYX‐008‐6.3‐12).

#### 2.3.2 Establishment of *S. aureus* ‐Induced Chronic Rhinosinusitis (CRS) Model

A chronic rhinosinusitis model was established using a *S. aureus* strain ATCC 25923 (American Type Culture Collection, Manassas, VA, USA).

Briefly, rats were anesthetized, and a sterile, silk‐suture‐tied expandable sponge (3 mm × 5 mm), saturated with 50 μL of *S. aureus* suspension (1.0 × 10^8^ CFU/mL), was carefully inserted into the left nasal cavity to a depth of approximately 15 mm from the anterior naris, positioning it at the sinonasal ostium complex. Subsequently, 500 μL of the same *S. aureus* suspension (1.0 × 10^8^ CFU/mL) was instilled into the left nasal cavity using a 1‐mL syringe.

To maintain the chronic infection, an additional 100 μL of *S. aureus* suspension (1.0 × 10^8^ CFU/mL) was administered intranasally to the left cavity twice per week for 3 weeks. Following each instillation, rats were suspended by their tails for 30–60 s to prevent aspiration and potential pulmonary infection. All procedures were performed aseptically within a biosafety cabinet. The modeling period concluded on Day 21.

The development of rhinosinusitis was monitored by observing symptoms such as nasal discharge and nose scratching. Clinical symptoms were evaluated according to a standardized scoring system for rhinosinusitis in rats, as previously described (Fang et al. 2025), with minor modifications (Table 1). A total symptom score > 5 on Day 21 was considered indicative of successful model establishment.

**TABLE 1:** Scoring criteria for the evaluation of rhinosinusitis symptoms in rats.

Symptom	Mild (Score 1)	Moderate (Score 2)	Severe (Score 3)
Nose rubbing (per 30 min)	1–4 times	5–9 times	More than 10 times
Sneezing (per 30 min)	1–4 times	5–9 times	More than 10 times
Nasal discharge	Slight runny nose	Runny nose to the nostrils	Secretions adhered around the nasal area
Nasal inflammation	Nasal congestion	Nasal swelling	Nasal swelling with congestion

Of the 60 rats subjected to the modeling procedure, all exhibited successful induction of rhinosinusitis (total symptom score > 5 on Day 21). Among these, 24 rats demonstrating the most severe and consistent symptoms (total score ≥ 8) were selected for the present PKP efficacy study. The remaining 36 rats with mild‐to‐moderate symptoms (total score 5–7) were allocated to a separate parallel study evaluating a different therapeutic agent, thereby maximizing the ethical use of animals in accordance with the 3R principles. This allocation strategy ensured a uniform and highly symptomatic cohort for the current investigation while minimizing overall animal usage.

#### 2.3.3 Dose Selection and Human Equivalent Dose (HED) Estimation

PKP at concentrations of 200, 400, and 600 mg/mL (administered in a volume of 100 μL per rat, equivalent to 20, 40, and 60 mg of PKP per administration, or approximately 88.9, 177.8, and 266.7 mg/kg, respectively) were selected for this in vivo study based on a comprehensive rationale that considered both efficacy and physiological relevance.

First, the dose range was preliminarily informed by our in vitro antibacterial results, which demonstrated significant, concentration‐dependent bactericidal activity against *S. aureus* , with an efficacy of 89.8% at 400 mg/mL (Figure 2B). To ensure therapeutic relevance, these effective in vitro concentrations were used as a starting point for the in vivo dosing strategy.

Second, and critically, to justify the physiological relevance of our chosen doses, we converted the animal dose to a human equivalent dose (HED) based on body surface area (BSA), which is the most widely accepted method for cross‐species dose translation (Jacob et al. 2022).

The calculation was performed as follows:

1. Rat dose calculation: The administered dose per rat was calculated. For the mid‐dose group (400 mg/mL, 100 μL), the total PKP delivered per administration was 40 mg. Considering an average rat body weight of 0.225 kg, the dose was 177.8 mg/kg.

Calculation: (400 mg/mL × 0.1 mL)/0.225 kg = 177.8 mg/kg.

2 HED calculation: The HED (in mg/kg) was estimated using the formula:
  HED (mg/kg) = animal dose (mg/kg) × (animal K_m/human K_m) (Reagan‐Shaw et al. 2008)
  where K_m is the body weight (kg) to body surface area (m^2^) conversion factor. The standard K_m values are 6 for the rat and 37 for a 60 kg human (Jacob et al. 2022).

Calculation: HED = 177.8 mg/kg × (6/37) ≈ 28.8 mg/kg.

3 Total human daily dose: For a 60 kg adult, this HED translates to a total daily dose of approximately 1.73 g of PKP.

Calculation: 28.8 mg/kg × 60 kg = 1728 mg (≈1.73 g).

Finally, we estimated the amount of the source herb required to yield this physiologically relevant dose. The extraction protocol (Section 2.1) yielded PKP at a rate of 17.96% (w/w) from the dried *Polygonatum kingianum* rhizome. Therefore, to obtain 1.73 g of PKP, the required amount of dried herb is approximately 9.6 g.

Calculation: 1.73 g PKP/0.1796 ≈ 9.6 g of dried herb.

This quantity of dried *Polygonatum kingianum* is well within the range of typical daily intakes used in traditional Chinese medicine and as a dietary supplement, thereby confirming the physiological relevance and translational potential of our experimentally administered doses.

#### 2.3.4 Experimental Design and Treatment Protocol

Upon successful model induction, rats were randomly divided into four groups (*n* = 6 per group) and one healthy group (*n* = 6) using a random number table method:

- Healthy group: Uninfected, healthy rats without any treatment.
- Model group: *S. aureus*‐infected rats treated daily with 100 μL of vehicle solution (e.g., saline or PBS) via intranasal instillation.
- PKP‐200 group: *S. aureus* ‐infected rats treated daily with 100 μL of PKP at 200 mg/mL.
- PKP‐400 group: *S. aureus*‐infected rats treated daily with 100 μL of PKP at 400 mg/mL.
- PKP‐600 group: *S. aureus* ‐infected rats treated daily with 100 μL of PKP at 600 mg/mL.

Treatments were administered once daily for 14 consecutive days, starting from the day after model confirmation (Day 22).

#### 2.3.5 Sample Collection and Processing

On the 14th day of treatment, 24 h after the final administration, all rats were deeply anesthetized with 0.2 mL/10 g of tribromoethanol for sample collection.

##### 2.3.5.1 Blood Collection and Analysis

Blood samples were collected from the orbital venous plexus. A portion was used for complete blood count (CBC) and biochemical analysis. The remaining blood was centrifuged at 3000 rpm for 10 min at 4°C to obtain serum, which was stored at −80°C for subsequent quantification of inflammatory cytokines. Serum IL‐6 levels were measured using a commercially available rat‐specific ELISA kit (Cat. No. JM‐01597R2; Jingmei Biotechnology, Jiangsu, China), and serum IL‐22 levels were measured using a rat‐specific ELISA kit (Cat. No. JM‐02076R2; Jingmei Biotechnology, Jiangsu, China), according to the manufacturers' protocols. All samples were analyzed in duplicate.

##### 2.3.5.2 Histopathological Examination

Following blood collection, animals were transcardially perfused with 200 mL of 2% paraformaldehyde in 0.1 M PBS for tissue fixation. The heads were decapitated and postfixed overnight. The skulls were then decalcified in ethylenediaminetetraacetic acid (EDTA) until soft. A coronal block from the plane posterior to the incisors to the plane anterior to the first molars was processed, dehydrated, and embedded in paraffin. Serial 5‐μm thick coronal sections were cut.

For histopathological evaluation, sections were stained with hematoxylin and eosin (H&E). Neutrophil infiltration in the sinus mucosa was quantified under light microscopy at 200× and 400× magnification and graded on a four‐point scale: (−) no neutrophils observed; (+) small aggregates of 1–10 neutrophils per high‐power field (HPF); (++) moderate infiltration of 11–30 neutrophils/HPF; (+++) severe focal infiltration of 31–90 neutrophils/HPF; (++++) extensive focal infiltration of > 90 neutrophils/HPF.

##### 2.3.5.3 Organ Weight and Pathological Observation

The spleen and thymus were carefully dissected and weighed immediately after extraction. The organ coefficient (organ‐to‐body weight ratio) was calculated for each animal.

#### 2.3.6 Statistical Analysis

Data are presented as the mean ± standard deviation. Statistical significance was determined by one‐way analysis of variance (ANOVA) followed by a post hoc test, such as Tukey's test. A *p* value of < 0.05 was considered statistically significant.

#### 2.3.7 Safety Evaluation of PKP in Healthy Rats

To exclude potential cytotoxicity, myelosuppression, and nonspecific immunomodulation by PKP, an additional cohort of healthy (non‐infected) adult male Sprague–Dawley rats (200–250 g) was employed. Animals were randomly divided into four groups (*n* = 6 per group) and treated daily for 14 consecutive days via intranasal instillation with either vehicle (Control) or PKP at 200, 400, and 600 mg/mL (100 μL per administration), following the identical dosing regimen described in Section 2.3.4. On Day 15 (24 h after the final administration), rats were deeply anesthetized and subjected to the same sample collection and processing protocols detailed in Section 2.3.5, including blood collection for complete blood count, serum cytokine quantification (IL‐6 and IL‐22), histopathological examination of nasal sinus mucosa, and dissection and weighing of the spleen and thymus. Statistical analysis was performed as described in Section 2.3.6.

## 3 Results and Discussion

### 3.1 Antimicrobial Activities In Vitro

To comprehensively characterize antibacterial potency, we first determined the MIC and MBC of PKP against *S. aureus* by 96‐well broth microdilution. The MIC was 400 mg/mL and the MBC was 700 mg/mL (MBC/MIC = 1.75), confirming bactericidal activity. We did not include 700 mg/mL in the subsequent plate‐counting validation for the following reasons: (1) the high‐concentration markedly increases solution viscosity, which interferes with uniform bacterial dispersion and accurate colony enumeration; (2) concentrations approaching 700 mg/mL are pharmacologically and economically impractical for topical formulation development; and (3) the selected range of 200–400 mg/mL already brackets the MIC and sufficiently captures the concentration‐dependent bactericidal trend.

Based on these benchmark values, antibacterial incubation experiments were performed by exposing *S. aureus* to PKP at 200, 300, and 400 mg/mL, covering concentrations below and at the MIC. For *E. coli* , PKP concentrations of 200–600 mg/mL were required, owing to its lower susceptibility. In these assays, PKP exhibited significant concentration‐dependent antibacterial activity against both strains. Previous studies have shown that the antibacterial and antioxidant attributes of plant extracts might be correlated with their total phenolic levels (Belew et al. 2025). Although PKP is a polysaccharide and not a phenolic compound, similar concentration‐dependent trends were observed here. In experiments against *S. aureus* , the bactericidal rates reached 73.2%, 82.6%, and 89.8% at PKP concentrations of 200, 300, and 400 mg/mL, respectively. Against *E. coli* , the bactericidal rates showed a gradual increasing trend within the concentration range of 200 to 600 mg/mL, specifically recording 27.6% (200 mg/mL), 47.7% (300 mg/mL), 54.7% (400 mg/mL), 62.5% (500 mg/mL), and 72.1% (600 mg/mL) (Figure 1A,B). The results indicated that PKP possesses certain inhibitory effects against both Gram‐positive bacteria (such as *S. aureus* ) and Gram‐negative bacteria (such as *E. coli* ), with consistently stronger activity against *S. aureus* . Although the specific mechanism underlying this differential efficacy was not directly examined in this study, several structural and physiological differences between Gram‐positive and Gram‐negative bacteria might explain this, as follows:

1. The outer membrane of Gram‐negative bacteria serves as a natural physical barrier, which may restrict the entry of macromolecular substances such as PKP, thereby preventing them from readily accessing their membrane targets. In contrast, Gram‐positive bacteria lack this barrier, making them more susceptible to macromolecular agents and allowing PKP to exert its effects more effectively (Silhavy et al. 2010).
2. Porins function as selective diffusion barriers for antibacterial agents in Gram‐negative bacteria. Negatively charged molecules, such as many natural polysaccharides, experience electrostatic repulsion with the negatively charged residues within the porin channels, further hindering their penetration. Consequently, PKP may be unable to efficiently traverse the porin channels of Gram‐negative bacteria due to its molecular size or charge characteristics, resulting in low intracellular concentrations and limited bioactivity (Pagès et al. 2008).
3. Gram‐negative bacteria contain highly efficient tripartite efflux pump systems (e.g., AcrAB‐TolC) with broad substrate specificity, encompassing numerous structurally unrelated hydrophobic and amphiphilic compounds. If PKP exhibits certain amphiphilic properties, it is highly likely to be recognized as a substrate by these efflux pumps and actively extruded, preventing its accumulation to effective concentrations within the cell (Li et al. 2015).
4. PKP may demonstrate higher binding affinity for specific components of the Gram‐positive bacterial cell membrane, such as teichoic acids, leading to stronger adhesion and more pronounced disruptive effects. In contrast, its interaction with lipopolysaccharides (LPS) of Gram‐negative bacteria is presumably weaker. Previous research has shown that plant‐derived polysaccharides exert antibacterial activity by disrupting the cell wall of Gram‐positive bacteria (Liang et al. 2024). It is therefore plausible to speculate that PKP may act through a similar membrane‐disrupting mechanism, although this requires direct experimental validation in future work.

**FIGURE 1:**
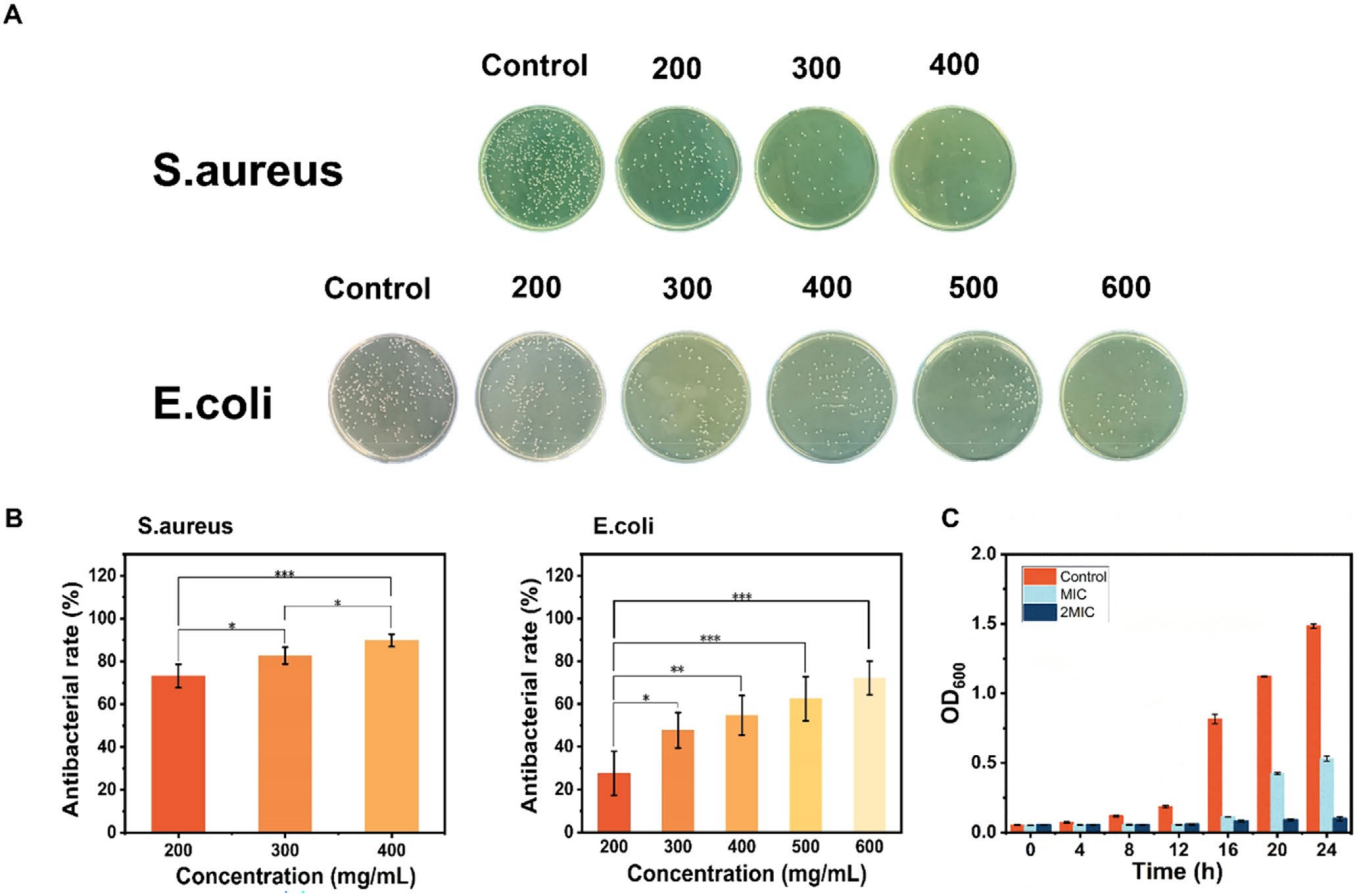
In vitro antibacterial activity of PKP against *Staphylococcus aureus* and *Escherichia coli* . (A) Representative images of bacterial growth inhibition for *S. aureus* and *E. coli* after treatment with different concentrations of PKP (200, 300, 400, 500, and 600 mg/mL). Control groups received no treatment. (B) Bar graph of the antibacterial rate of PKP against *S. aureus* (200, 300, and 400 mg/mL) and *E. coli* (200, 300, 400, 500, and 600 mg/mL). *, ** and *** indicate *p* < 0.05, *p* < 0.01 and *p* < 0.001. (C) Representative time‐kill curves of *S. aureus* after treatment with PKP at MIC (400 mg/mL) and 2 × MIC (800 mg/mL). Bacterial growth was monitored by OD_600_ measurements at 4‐h intervals over 24 h. Control groups received no PKP. Data are presented as mean ± SD (*n* = 3).

Together, these findings demonstrate that PKP, as a natural product, has potential application value in inhibiting common pathogenic bacteria, particularly showing more significant inhibitory effects against Gram‐positive bacteria. These findings provide experimental evidence for its development in fields such as food preservation or pharmaceuticals.

It should be acknowledged that the antibacterial assessment in this study was limited to single reference strains of *S. aureus* and *E. coli* . Future studies incorporating multiple clinical isolates of these species, as well as additional clinically relevant Gram‐positive and Gram‐negative bacteria (e.g., *Staphylococcus epidermidis* , *Streptococcus pyogenes* , *Streptococcus pneumoniae* , *Enterococcus faecalis* , *Klebsiella pneumoniae* , and different lineages of *E. coli* ), will be essential to fully define the spectrum of activity and intraspecies variability of PKP susceptibility. Such expanded strain panels would provide critical insight into whether the observed differential efficacy between Gram‐positive and Gram‐negative organisms is a consistent feature of PKP or subject to strain‐specific variation.

To further characterize the bactericidal kinetics of PKP, a time‐kill assay was performed by monitoring *S. aureus* growth over 24 h in the presence of PKP at MIC (400 mg/mL) and 2 × MIC (800 mg/mL) (Figure 1C). The control culture exhibited a typical growth curve: OD_600_ remained near baseline during the first 8 h (0.054–0.118), entered early logarithmic growth by 12 h (0.185), and then underwent rapid proliferation between 16 and 20 h (0.814–1.122), approaching stationary phase by 24 h (1.484). In contrast, cultures treated with PKP at MIC showed complete growth suppression for the first 12 h (OD_600_ 0.051–0.055), followed by a modest resurgence at 16 h (0.113) and gradual increase to 0.424 at 20 h and 0.530 at 24 h. This pattern indicates that PKP at MIC exerts a primarily bacteriostatic effect during the initial 12 h, but its inhibitory potency gradually wanes thereafter, allowing sublethal bacterial populations to resume proliferation at a suppressed rate.

At 2 × MIC (800 mg/mL), PKP completely abolished bacterial growth throughout the entire 24‐h observation period. OD_600_ values remained at baseline levels for the first 12 h (0.053–0.057) and showed only marginal elevation at 16 h (0.081), 20 h (0.091), and 24 h (0.098), with no evidence of regrowth. This near‐total suppression of turbidity is consistent with rapid bactericidal activity, in which bacterial cells are killed or rendered nonviable shortly after exposure, precluding any measurable increase in biomass.

These time‐kill data complement the MIC/MBC determination, where the MBC (700 mg/mL) was approximately 1.75‐fold the MIC, classifying PKP as a bactericidal agent. The kinetic profile reveals, however, that this bactericidal activity is sharply concentration‐dependent: at MIC, the effect is transiently bacteriostatic, with bacterial regrowth evident after 16 h; at 2 × MIC, killing is sustained and complete. This concentration‐dependent acceleration is characteristic of membrane‐active antimicrobial agents, for which higher concentrations achieve faster and more extensive membrane disruption, leading to rapid cell death. The fructan‐rich structure of PKP, with its high molecular weight and potential amphiphilic character, may facilitate interaction with the *S. aureus* cell envelope, progressively compromising membrane integrity as concentration increases.

Finally, we acknowledge an important interpretive limitation regarding the high MIC value (400 mg/mL). At this concentration range, the elevated solution viscosity and osmotic pressure of the polysaccharide solution may contribute to apparent bacterial growth suppression, independent of specific antimicrobial action. Although the MBC/MIC ratio of 1.75 and the concentration‐dependent acceleration of killing observed in the time‐kill assay support a genuine bactericidal mechanism, we cannot entirely exclude the possibility that nonspecific physical effects partially contributed to the observed antibacterial activity. Future studies incorporating an inert high‐molecular‐weight polysaccharide (e.g., dextran) as an osmotic and viscosity control would be necessary to formally delineate the specific antimicrobial contribution of PKP from these confounding physical factors.

### 3.2 Antibiofilm Activity of PKP

To evaluate whether PKP could disrupt established *S. aureus* biofilms, mature biofilms were exposed to PKP at 1/2 MIC, MIC, and 2 × MIC for 48 h. Crystal violet staining revealed a clear concentration‐dependent reduction in biofilm biomass (Figure 2). Control wells retained dense, uniformly purple‐stained biofilms covering the entire well surface, corresponding to OD_570_ values of 1.92 and 1.58 (mean 1.75). In contrast, wells treated with 1/2 MIC PKP (200 mg/mL) showed visibly attenuated staining intensity, with OD_570_ values of 1.32 and 1.27, representing 74.0% residual biomass relative to the control. At the MIC (400 mg/mL), biofilm disruption was more pronounced: staining appeared patchy and markedly lighter, and OD_570_ dropped to 0.96 and 0.88 (mean 0.92, 52.6% of control). The most remarkable effect was observed at 2 × MIC (800 mg/mL), where the crystal violet signal was barely detectable in most areas of the well, with OD_570_ values of 0.32 and 0.14 (mean 0.23, 13.1% of control), indicating near‐complete eradication of the established biofilm.

**FIGURE 2:**
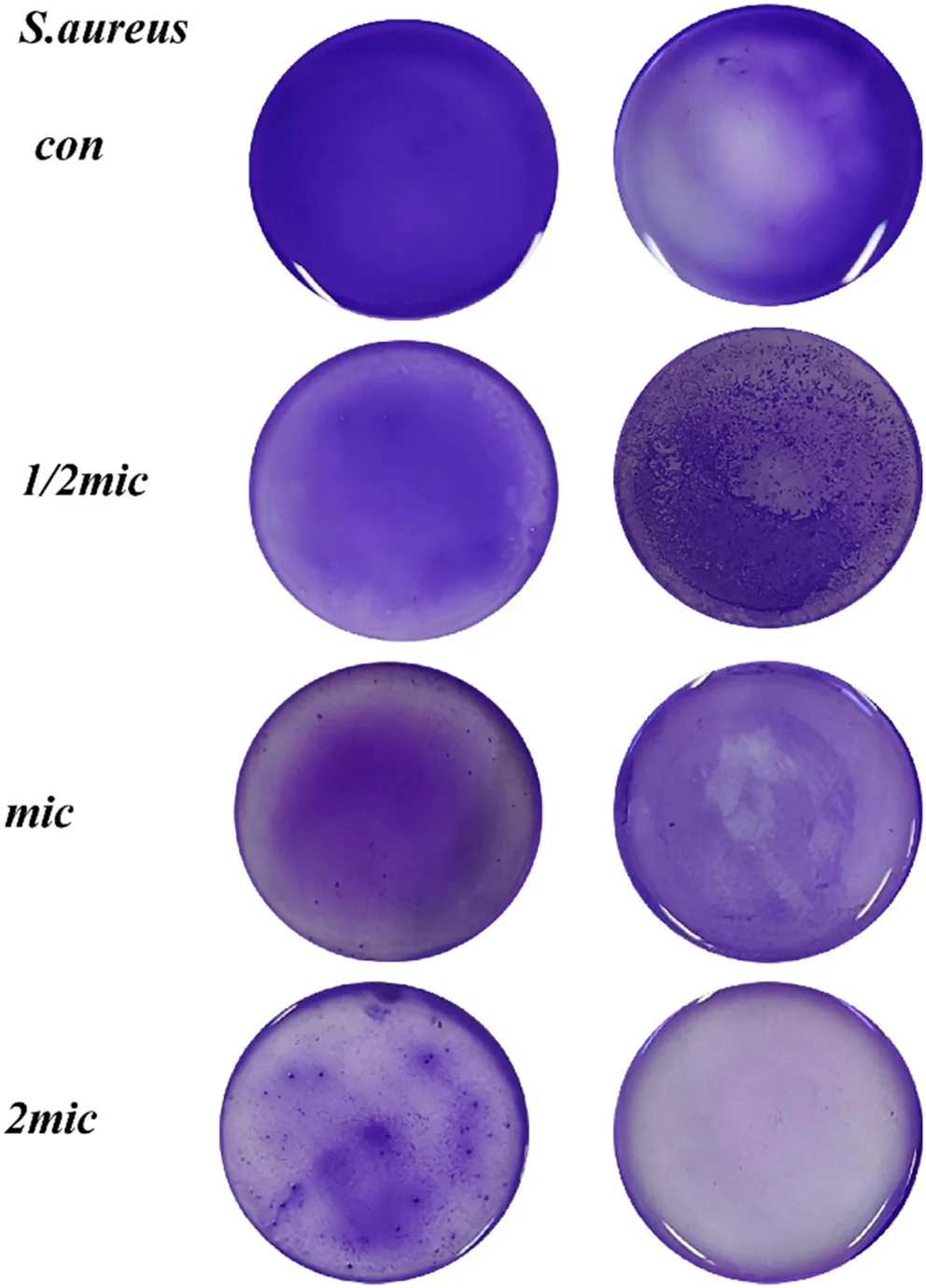
Crystal violet staining of established *Staphylococcus aureus* biofilms after 48‐h exposure to PKP. Con, control (no PKP); 1/2 MIC, 1/2 × MIC (200 mg/mL); MIC, 1 × MIC (400 mg/mL); 2 × MIC, 2 × MIC (800 mg/mL). Each group shows two replicate wells.

These results demonstrate that PKP possesses potent antibiofilm activity against *S. aureus* in a concentration‐dependent manner. Notably, even at the MIC (400 mg/mL), which was previously determined against planktonic bacteria in suspension, PKP reduced established biofilm biomass by nearly half. This is significant because biofilm‐encased bacteria typically exhibit 10‐ to 1000‐fold increased tolerance to antimicrobial agents compared to their planktonic counterparts, often necessitating concentrations far exceeding the MIC to achieve comparable killing (Sauer et al. 2022). The heightened recalcitrance stems from the extracellular polymeric substance matrix, persister cell populations, and metabolic dormancy that collectively shield embedded bacteria from both antibiotic penetration and host immune clearance. The fact that PKP at its MIC could substantially disrupt a 36‐h pre‐formed biofilm suggests that the polysaccharide may penetrate the extracellular polymeric substance (EPS) matrix (Liu et al. 2024; Zong et al. 2023) or interfere with the adhesion‐related molecules that mediate bacterial attachment to the substratum (Xu et al. 2022).

The near‐complete clearance observed at 2 × MIC (800 mg/mL) is particularly noteworthy. At this concentration, PKP likely operates through multiple mechanisms. First, as a high‐molecular‐weight fructan‐rich polysaccharide, PKP may physically disrupt the EPS architecture by competing for binding sites or altering the hydration status of the biofilm matrix, thereby promoting the detachment of bacteria from the biofilm matrix (Pinto et al. 2020). Second, given the direct bactericidal activity demonstrated against planktonic *S. aureus* (Section 3.1), PKP may penetrate the biofilm and exert bactericidal effects on biofilm‐encased bacteria (Didi et al. 2019), consistent with recent observations that established biofilms can be eradicated by agents that disrupt both the extracellular matrix and the metabolic dormancy of persister cells (Rowe et al. 2021). Third, the reduction in biomass at 1/2 MIC (74.0% residual) suggests that subinhibitory concentrations of PKP can still impair biofilm maintenance.

The disparity in efficacy between 1/2 MIC and 2 × MIC highlights a steep concentration‐response relationship for biofilm eradication. While 200 mg/mL merely attenuated biofilm density, doubling the dose to 400 mg/mL (MIC) achieved a quantitatively larger reduction, and a further doubling to 800 mg/mL essentially eliminated the biofilm. This steepness suggests that PKP's antibiofilm effect is not merely a linear extension of its bacteriostatic activity but may involve a threshold‐dependent mechanism (Lu et al. 2024)—such as EPS saturation or membrane disruption—that is triggered once a critical polysaccharide concentration is reached within the biofilm microenvironment.

From a translational perspective, these findings are highly relevant to the pathophysiology of chronic rhinosinusitis, where *S. aureus* biofilms on sinonasal mucosa act as persistent reservoirs that evade both immune clearance and conventional antibiotic therapy (Chegini et al. 2023; Sauer et al. 2022). The ability of PKP to eradicate established biofilms at concentrations that are pharmacologically achievable in topical delivery settings—particularly given its natural origin and favorable safety profile—positions it as a promising candidate for biofilm‐associated upper respiratory infections. Future studies should employ confocal laser scanning microscopy with LIVE/DEAD staining to visualize the spatial distribution of PKP within the biofilm architecture and to confirm whether the observed biomass reduction corresponds to bacterial death or merely to biofilm dispersal without lethal effects.

### 3.3 Antibacterial Activity and Eradication of *S. aureus* Infection In Vivo

Given the notable antibacterial activity of PKP in vitro, it is necessary to determine whether bacterial infection can be effectively eliminated in the complex biological environment. Therefore, we evaluated the in vivo antibacterial efficacy of PKP using a rat model of *S. aureus* ‐induced rhinosinusitis. To evaluate the outcome of the *S. aureus* rhinosinusitis model in rats, each symptom was graded on a scale of 0–3 (0: none, 1: mild, 2: moderate, 3: severe) according to the criteria detailed in Table 1. A total symptom score (the sum of all four individual scores) > 5 on Day 21 was considered indicative of successful model establishment.

Rats meeting this criterion displayed pronounced rhinosinusitis symptoms, typically including frequent nose rubbing (> 5 times/30 min), repeated sneezing episodes (> 5 times/30 min), runny nose extending to the nostrils or with secretions adhered around the nasal area, and evident nasal swelling with congestion. From the pool of 60 successfully modeled rats, a total of 24 animals that demonstrated the most consistent and high symptom scores were selected. The relatively low proportion of rats (24/60) allocated to the PKP treatment group reflects a deliberate selection of the most severely affected animals to maximize the likelihood of detecting therapeutic efficacy, rather than a low modeling success rate. All 60 animals were successfully modeled, and the remaining 36 rats were utilized in a concurrent study, ensuring full compliance with ethical principles of reduction and refinement in animal research. These 24 rats were then randomly allocated into the experimental groups to ensure a uniform and highly symptomatic population for the subsequent investigations. Different concentrations of PKP were administered intranasally once a day. Animals were treated and monitored for 14 days and completed the hematological tests and dissection on the last day (Figure 3A).

**FIGURE 3:**
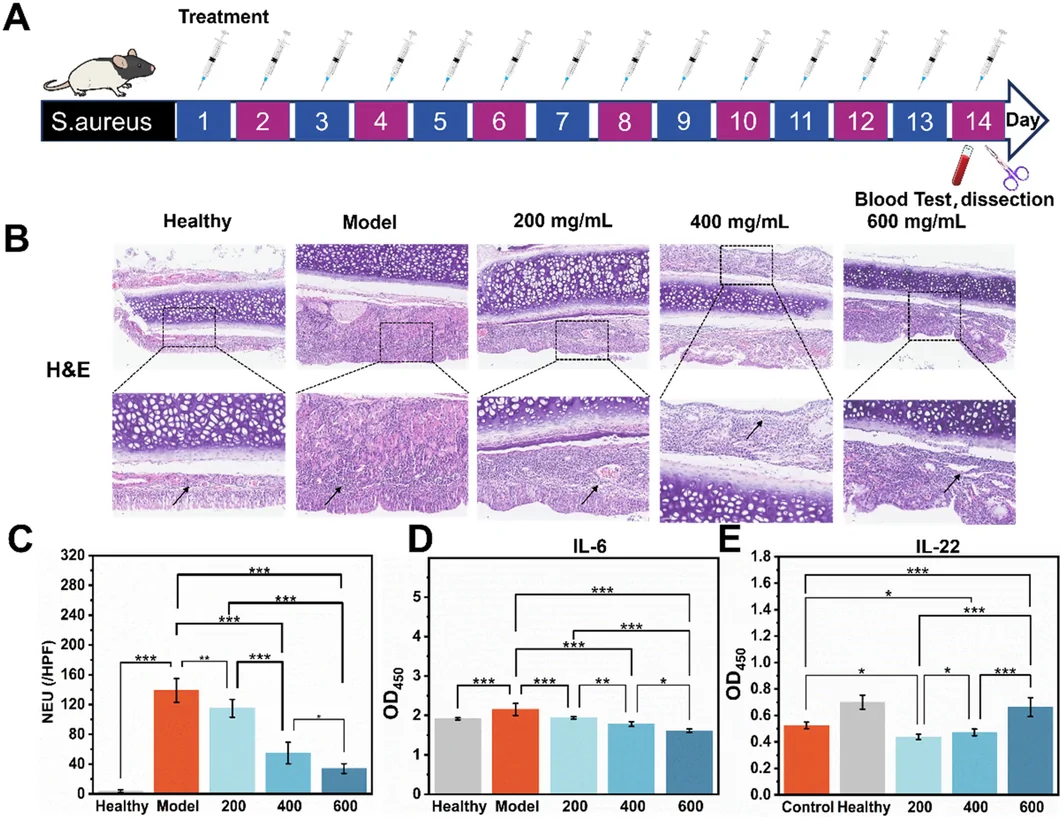
Evaluation of the therapeutic effect of PKP on rhinosinusitis in *Staphylococcus aureus* ‐infected rats. (A) Schematic diagram of establishment and treatment of *S. aureus* ‐infected rats rhinosinusitis model. (B) Representative histological H&E staining of *S. aureus* ‐infected rats 14 days after treatment in different treatment groups (Healthy, Model, 200, 400, and 600 mg/mL PKP), where black arrows indicate inflammatory exudate and necrotic tissue. (C) Maximal quantitative assessment of neutrophilic infiltration in the sinus mucosa of *S. aureus* ‐infected rats following 14‐day treatment with PKP. (D, E) IL‐6 and IL‐22 levels in *S. aureus* ‐infected rats on Day 14 after treatment. Data are represented as the mean ± SD (*n* = 6). Error bars indicate the SD. *, ** and *** indicate *p* < 0.05, *p* < 0.01 and *p* < 0.001, respectively, compared to the model control (0 mg/mL PKP) group. ns means no statistical difference (*p* > 0.05).

Subsequently, sinus cavity mucosa samples were stained with hematoxylin and eosin (H&E) for histological examination (Figure 3B). After 14 days of treatment, the sinus cavity mucosal tissues of the model and 200 mg/mL PKP‐treated groups exhibited pronounced neutrophilic infiltration, with focal accumulations observed in both the mucosal layer and interstitium, reaching an average number of 139 and 115 neutrophils per high‐power field (HPF) in the model group and 200 mg/mL PKP‐treated groups respectively (Figure 3C). Additionally, fibrous tissue hyperplasia, focal necrosis, and apparent abscess formation were detected. In contrast, the 400 mg/mL PKP‐treated group (showing focal neutrophil aggregation with an average count of 55/HPF) and the 600 mg/mL PKP‐treated group (exhibiting focal neutrophil accumulation with an average count of 34/HPF) demonstrated markedly reduced neutrophilic infiltration compared to the control group. Abscess formation was absent in both high‐concentration treatment groups. These findings indicate that higher concentrations of PKP attenuate sinusitis severity in rats, reduce neutrophil aggregation, suppress inflammatory severity, and confer a protective effect.

This view is also supported by the results of enzyme‐linked immunosorbent assay (ELISA) (Figure 3D,E). Serum IL‐6 levels (Figure 3D), a key mediator of systemic inflammation, were significantly reduced in the 600 mg/mL PKP group compared with all other groups. Our results indicate that IL‐6 contributes to systemic inflammation and can be effectively reduced by treatment with high concentration of PKP. IL‐6 is a key driver of neutrophilic inflammation and tissue remodeling in chronic rhinosinusitis, and its sustained elevation has been linked to disease severity and polypoid tissue changes (Cui et al. 2023; Petalas et al. 2023). This suggests that higher concentrations of PKP treatment, by effectively reducing IL‐6, may impede the recurrence of rhinosinusitis associated with persistent mucosal inflammation.

To more comprehensively evaluate the therapeutic effect of PKP on rhinosinusitis, we investigated the serum levels of IL‐22 in each group (Figure 3E). Compared with healthy controls, rats in the model group showed a marked decrease in IL‐22, consistent with impaired epithelial barrier function during infection. PKP treatment at 200, 400, and 600 mg/mL resulted in a dose‐dependent increase in IL‐22 levels compared to the model group, with the 600 mg/mL group achieving values comparable to the healthy group. Notably, IL‐22 levels in the 200 and 400 mg/mL groups remained below those of healthy controls, suggesting that complete restoration of barrier‐protective function requires higher doses of PKP. IL‐22 is a critical cytokine for mucosal barrier maintenance, promoting epithelial repair and limiting excessive immune activation by enhancing tight junction integrity and stimulating antimicrobial peptide production (Kang et al. 2025). By restoring epithelial barrier function, IL‐22 may indirectly reduce leukocyte infiltration into inflamed tissues, thereby attenuating the amplification of pro‐inflammatory immune responses driven by pathogen‐associated molecular patterns and tissue damage signals. This observation elucidated the significant reduction in IL‐22 levels observed in rats infected with *S*. *aureus* compared to healthy controls, followed by an elevation after PKP treatment. This phenomenon suggested that PKP may facilitate the restoration of immune barrier function following infection and dampen inflammatory amplification during infection.

It should be acknowledged that, while the in vivo efficacy of PKP was strongly supported by histopathological amelioration, cytokine modulation, and hematological normalization, direct quantification of residual bacterial load (e.g., CFU counts from sinus mucosal homogenates) was not performed in the present study. Consequently, we refrain from making definitive claims regarding bacterial eradication, and instead interpret the observed therapeutic benefits as reflecting a combined direct antibacterial and host‐mediated immunomodulatory effect. Future studies incorporating serial bacterial load measurements would be essential to conclusively establish the bactericidal potency of PKP in complex physiological environments and to delineate the relative contribution of pathogen clearance versus host inflammatory modulation.

### 3.4 Hematological Profile

In a rat model of *S. aureus* ‐induced sinusitis, administration of PKP was evaluated through hematological analysis of leukocytes, lymphocytes, neutrophils, cholesterol, and immune organ indices. The experimental groups included: a model group (untreated infected rats), a healthy group (uninfected rats), and three PKP‐treated groups (200, 400, and 600 mg/mL).

Compared with the model group (11.47 ± 2.55 × 10^9^/L), PKP treatment significantly reduced leukocyte counts (6.46 ± 1.26–6.62 ± 1.70 × 10^9^/L), indicating suppression of inflammatory cell infiltration and mitigation of systemic inflammation (Figure 4A). Beyond general leukocyte reduction, PKP also exerted a targeted effect on specific immune cell populations. Lymphocyte levels decreased markedly in PKP‐treated groups (4.28 ± 0.77–4.63 ± 1.54 × 10^9^/L) versus the model group (9.23 ± 2.42 × 10^9^/L), suggesting modulation of adaptive immunity and prevention of excessive immune activation (Figure 4B). In addition to lymphocytes, neutrophils—key mediators of acute inflammation—were also influenced by PKP. A notable reduction in neutrophils was observed in the 200 mg/mL group (1.13 ± 0.19 × 10^9^/L vs. 1.78 ± 0.29 in the model group), implying that PKP may alleviate acute mucosal inflammation by inhibiting inflammatory mediators (Figure 4C). Beyond its effects on immune cells, PKP also impacted systemic metabolic parameters. PKP induced a dose‐dependent decrease in total cholesterol (1.31 ± 0.19–1.43 ± 0.12 mmol/L vs. 1.50 ± 0.06 mmol/L in the model group), suggesting a potential modulatory effect on lipid metabolism. The exact mechanism underlying this effect warrants further investigation (Figure 4D). The immunomodulatory effects of PKP were further reflected in changes in immune organ indices: PKP increased the spleen index (0.23 ± 0.01–0.23 ± 0.03 vs. 0.19 ± 0.02 in the model group), possibly reflecting enhanced immune cell activity and pathogen clearance (Figure 4E). A dose‐dependent rise in thymus index (0.116–0.134 vs. 0.103) suggests improved T cell development and strengthened systemic immunity (Figure 4F).

**FIGURE 4:**
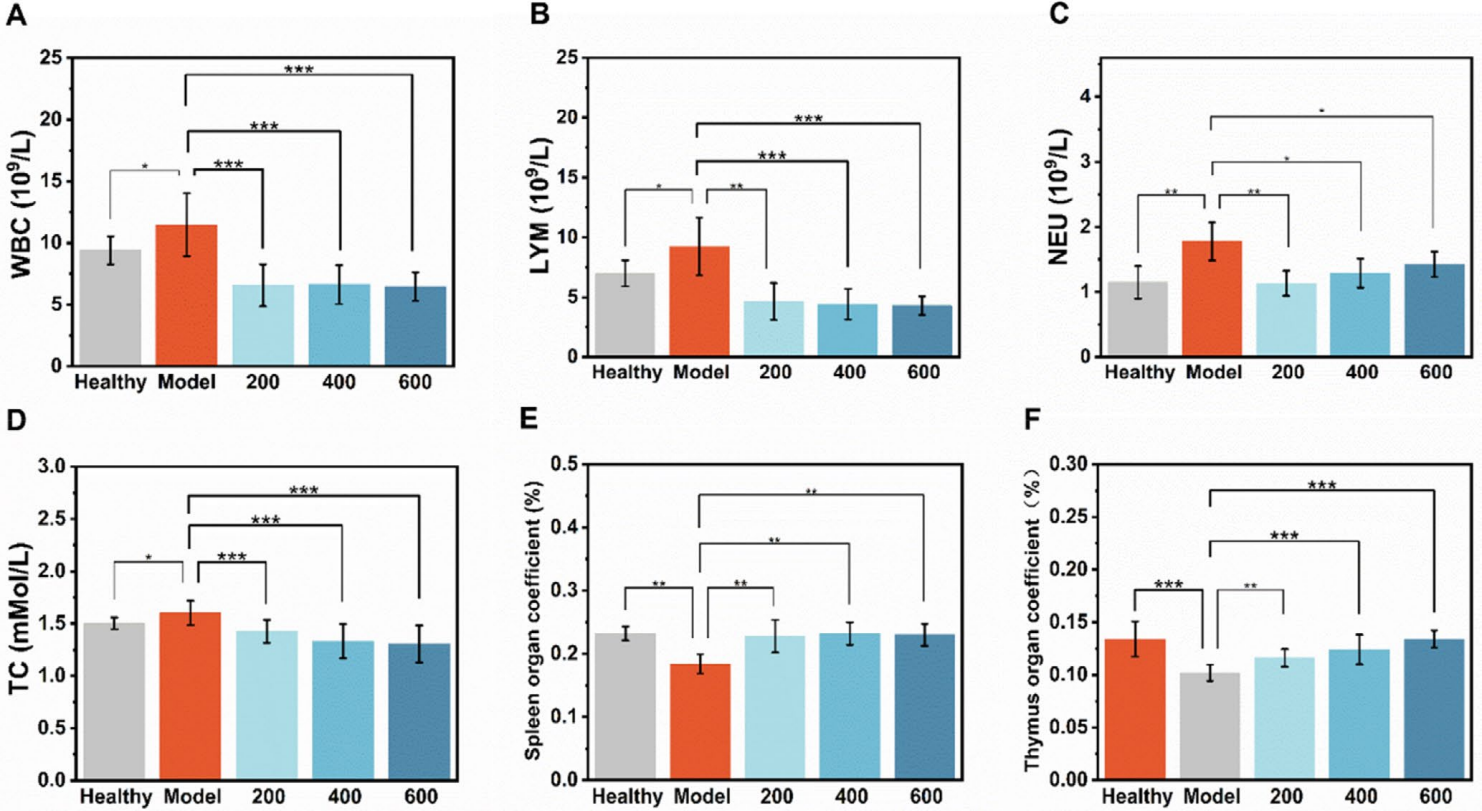
Hematological profile in animals of *Staphylococcus aureus* sinusitis following intranasal administration of PKP in vivo. (A) Total leukocyte count. (B) Lymphocyte count. (C) Neutrophil count. (D) Total cholesterol level. (E) Spleen index (spleen‐to‐body weight ratio). (F) Thymus index (thymus‐to‐body weight ratio). Data are presented as mean ± SD (*n* = 6). *, ** and *** indicate *p* < 0.05, *p* < 0.01, and *p* < 0.001 respectively, compared to the model group.

In summary, PKP modulated immune cell responses, enhanced immune organ function, and improved lipid metabolism. These combined effects contribute to the reduction of sinusitis‐related inflammation and may help mitigate long‐term risks associated with chronic inflammatory and metabolic disturbances.

### 3.5 Effects of PKP on Healthy Rats Without Bacterial Infection

To better distinguish host immunomodulation from the direct antimicrobial activity to the therapeutic efficacy observed in the infected model, and to exclude potential cytotoxicity or myelosuppressive effects of PKP on immune cells. we performed an additional in vivo control experiment in which healthy (non‐infected) rats received the same intranasal PKP regimens (200, 400, and 600 mg/mL) for 14 consecutive days.

Histopathological examination of the nasal sinus mucosa revealed intact epithelial architecture in all groups, with only a minimal number of scattered neutrophils (5/HPF) observed occasionally in the submucosa. No inflammatory cell infiltration, mucosal ulceration, abscess formation, or necrotic tissue was detected in either the control group or any PKP‐treated group, indicating that intranasal PKP does not induce local mucosal irritation or sterile inflammation in the absence of bacterial infection (Figure 5A).

**FIGURE 5:**
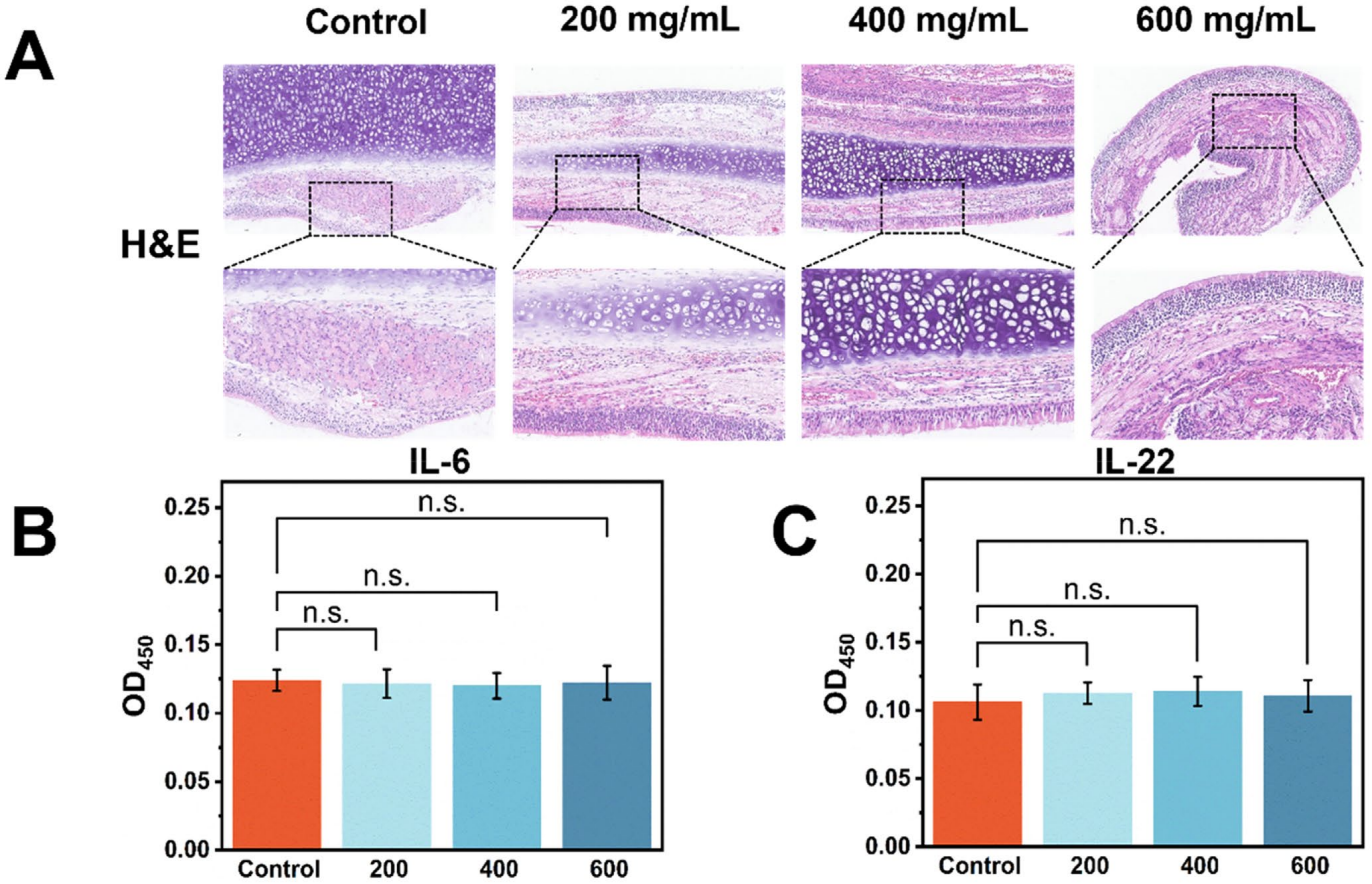
Evaluation of intranasal PKP administration in healthy rats. (A) Representative histological H&E staining of nasal sinus mucosa from healthy rats following 14‐day daily intranasal instillation of vehicle (Control) or PKP at 200, 400, and 600 mg/mL. Minute quantities of neutrophils (5/HPF); no mucosal ulceration or necrotic tissue was observed in the treatment group. (B) Serum IL‐6 levels and (C) serum IL‐22 levels in healthy rats on Day 14 after treatment. Data are represented as the mean ± SD (*n* = 6), where ns indicates no statistical difference (*p* > 0.05) among all groups by one‐way ANOVA followed by Tukey's post hoc test.

Collectively, these data establish that PKP does not exert nonspecific immunostimulatory or immunosuppressive effects in healthy animals. The absence of basal cytokine modulation in the healthy state (Figure 5B,C), combined with the restoration of pathologically altered IL‐6 and IL‐22 levels observed in the infected model (Figure 3D,E), supports a context‐dependent immunomodulatory mechanism rather than a direct, universal immune cell activation or cytotoxicity. Thus, the anti‐inflammatory and immune‐restorative effects of PKP in the *S. aureus* rhinosinusitis model are attributable to the modulation of dysregulated host immune responses during active infection, acting in concert with its direct antibacterial activity.

To exclude potential myelosuppression or lymphocytotoxicity, healthy rats (without *S. aureus* infection) were administered PKP intranasally at 200, 400, and 600 mg/mL for 14 consecutive days. Complete blood count analysis revealed that PKP did not significantly alter total leukocyte, lymphocyte, erythrocyte, hemoglobin, or platelet counts compared to the vehicle‐treated control (Table 2) (all *p* > 0.05; Figure 6A,B,D–F). The absence of statistical significance indicates that PKP exposure did not interfere with erythropoiesis or thrombopoiesis, nor did it induce nonspecific activation or suppression of circulating immune cells under basal conditions.

**TABLE 2:** Hematological parameters in healthy rats following 14‐day intranasal administration of vehicle (Control) or PKP at 200, 400, and 600 mg/mL.

	Control	PKP‐200	PKP‐400	PKP‐600
WBC (×10^9^/L)	8.44 ± 2.30	10.16 ± 2.44	8.58 ± 1.86	8.52 ± 1.21
NEU (×10^9^/L)	1.05 ± 0.24	2.29 ± 1.66	1.42 ± 0.32	2.20 ± 0.47
LYM (×10^9^/L)	6.87 ± 2.02	7.25 ± 1.23	6.61 ± 1.82	5.77 ± 1.21
RBC (×10^12^/L)	6.43 ± 0.34	6.24 ± 0.13	6.18 ± 0.10	6.36 ± 0.33
HGB (g/L)	128 ± 4.29	124 ± 3.44	124 ± 2.77	127 ± 4.09
PLT (×10^9^/L)	1118 ± 131	1226 ± 174	1096 ± 109	1187 ± 72

**FIGURE 6:**
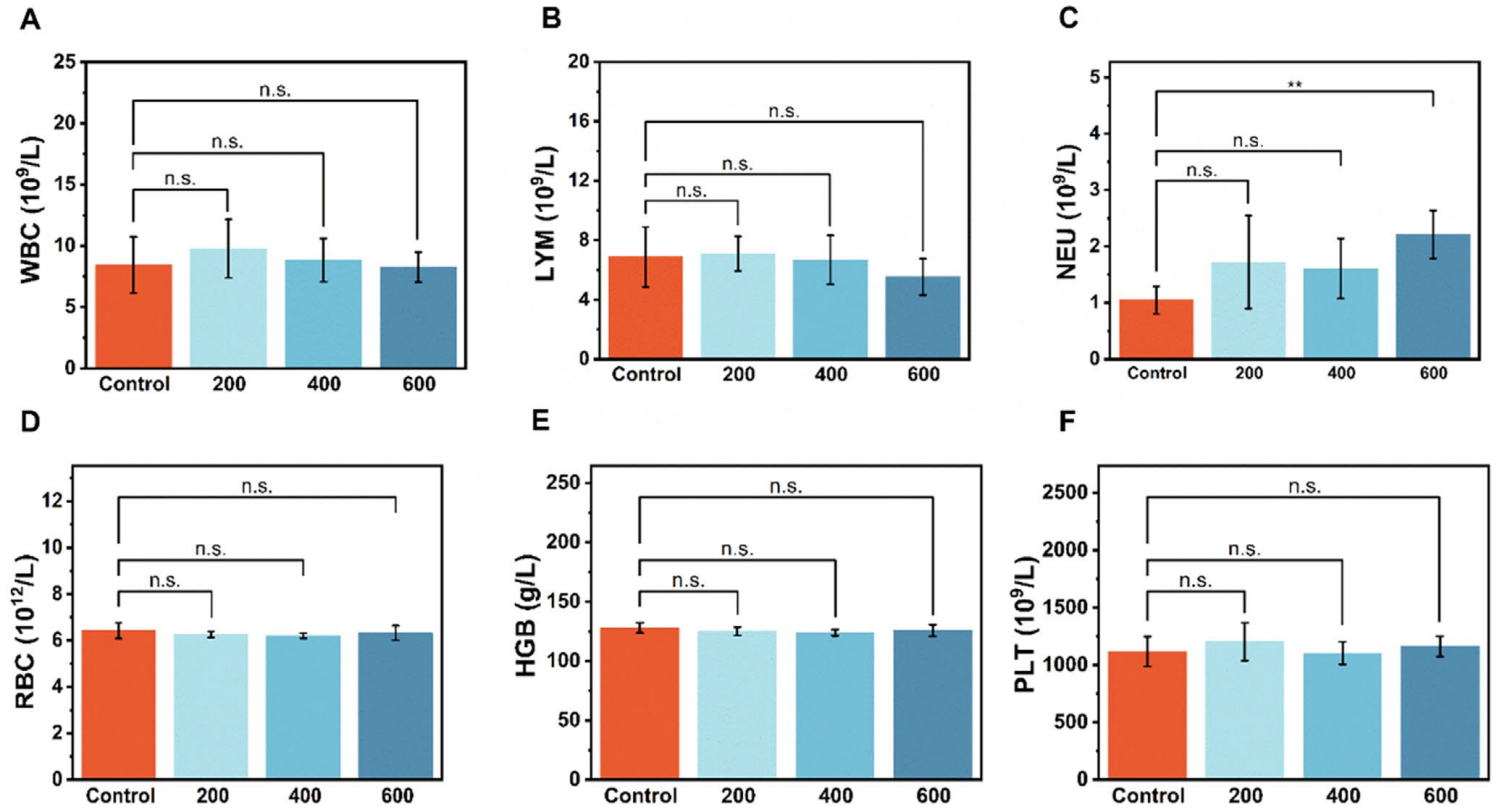
Hematological profile in healthy animals following intranasal administration of PKP in vivo. (A) Total leukocyte count. (B) Lymphocyte count. (C) Neutrophil count. (D) Erythrocyte level. (E) Hemoglobin level. (F) Platelet level. Data are presented as mean ± SD (*n* = 6). Statistical significance was determined by one‐way ANOVA followed by Tukey's post hoc test. For neutrophil counts (C), the PKP‐600 group was significantly different from the control group (**p < 0.01); all other pairwise comparisons in (C) and all comparisons in (A, B, D–F) were not significant (ns, *p* > 0.05).

Notably, although neutrophil counts in the PKP‐600 group (2.20 ± 0.47 × 10^9^/L) were statistically higher than those in the control group (1.05 ± 0.24 × 10^9^/L; **p < 0.01, Figure 6C). Whereas the PKP‐200 and PKP‐400 groups showed no significant difference compared to control. Nevertheless, all neutrophil counts fell well within the normal physiological range for Sprague–Dawley rats (typically 0.5–3.0 × 10^9^/L). Moreover, the PKP‐200 group exhibited a comparable elevation (2.29 ± 1.66 × 10^9^/L) that did not reach statistical significance owing to greater interindividual variance, and no significant differences were detected among the three PKP‐treated groups. Thus, the isolated statistical significance between control and PKP‐600—in the absence of a dose‐dependent trend across the three PKP groups—likely reflects baseline biological variability rather than a biologically relevant neutrophilia or inflammatory activation.

Taken together, the consistent absence of statistically significant differences across the majority of hematological indices, combined with the fact that all measured parameters remained within normal reference ranges, indicates that PKP does not perturb baseline hematopoietic homeostasis or exert cytotoxic effects on circulating immune cells in healthy animals.

Consistent with the absence of mucosal toxicity, hematological analysis of healthy rats receiving PKP revealed no significant alterations in total leukocyte, neutrophil, lymphocyte, erythrocyte, hemoglobin, or platelet counts compared to the uninfected control. Notably, whereas PKP treatment significantly reduced pathologically elevated WBC and neutrophil counts in infected rats toward baseline, it did not suppress these parameters below normal levels in healthy animals. Together with the restored immune organ indices observed in infected rats, these data confirm that PKP does not exert myelosuppressive or lymphocytotoxic effects, but instead selectively modulates dysregulated immune responses during active infection.

## 4 Conclusion

The escalating global burden of antimicrobial resistance, exemplified by recalcitrant *S. aureus* infections in chronic rhinosinusitis, necessitates the urgent development of alternative therapeutics that operate through mechanisms distinct from those of conventional antibiotics. This study provides comprehensive preclinical evidence that *Polygonatum kingianum polysaccharide* (PKP) fulfills this need through a dual mechanism of action: direct bactericidal activity against planktonic and biofilm‐encased *S. aureus* , coupled with context‐dependent host immunomodulation that restores dysregulated inflammatory and barrier‐protective responses without perturbing basal immune homeostasis.

In vitro, PKP exhibited concentration‐dependent bactericidal activity with a clear preference for Gram‐positive organisms, achieving an 89.8% killing rate against *S. aureus* at its MIC (400 mg/mL). The MBC/MIC ratio of 1.75 confirmed its bactericidal nature. Notably, PKP also demonstrated antibiofilm efficacy, reducing established *S. aureus* biofilm biomass by nearly 50% at the MIC and achieving near‐complete eradication at 2 × MIC. Given that biofilm persistence is a principal driver of chronicity and antibiotic failure in rhinosinusitis, this antibiofilm capacity represents a particularly valuable therapeutic attribute.

In a physiologically relevant rat model of *S. aureus* ‐induced chronic rhinosinusitis, intranasal PKP administration markedly attenuated disease severity, as evidenced by reduced neutrophilic infiltration, abscess resolution, and amelioration of mucosal pathology. These histopathological improvements were paralleled by rebalancing of key cytokine networks: PKP dose‐dependently suppressed the pro‐inflammatory mediator IL‐6 while restoring the barrier‐protective cytokine IL‐22 toward healthy baseline levels. Hematological and immunological profiling further corroborated these findings, revealing normalization of leukocyte and neutrophil counts alongside enhanced spleen and thymus indices—indicators of restored systemic immune competence.

Critically, an additional control experiment in healthy, non‐infected rats demonstrated that PKP at equivalent doses did not alter basal serum IL‐6 or IL‐22 levels, induce mucosal irritation, or disrupt hematopoietic homeostasis. This absence of effect in the healthy state, contrasted with its pronounced immunomodulatory efficacy during active infection, establishes that PKP does not act as a nonspecific immunosuppressant or immunostimulant. Rather, it exerts context‐dependent immunomodulation, selectively targeting pathologically dysregulated immune responses while sparing normal physiological function. This safety profile, combined with its natural origin and the translational relevance of the intranasal delivery route, positions PKP as a promising candidate for topical or adjunctive therapy in biofilm‐associated upper respiratory infections.

Collectively, these findings bridge a critical translational gap between in vitro bioactivity and in vivo efficacy, offering new mechanistic insights into how plant‐derived polysaccharides can function through the integrated actions of direct pathogen elimination and host‐directed immune restoration. Future studies should prioritize direct bacterial enumeration in infected tissues to quantitatively delineate the relative contributions of antimicrobial killing versus immunomodulation, optimize formulation and dosing regimens for clinical translation, and elucidate the precise molecular pathways—such as membrane disruption dynamics and cytokine signaling cascades—underlying PKP's dual mechanism. Exploration of PKP's efficacy against multidrug‐resistant clinical isolates and in combination with conventional antibiotics may further accelerate its development as a natural therapeutic agent for bacterial rhinosinusitis and related recalcitrant infections.

## Author Contributions

**Xu‐Chen Xia:** investigation, writing – original draft, formal analysis, data curation. **Ya‐Qi Peng:** writing – review and editing, funding acquisition, conceptualization, formal analysis. **Lu‐Xuan Liu:** data curation, formal analysis. **Bing Cao:** data curation, formal analysis. **Xue‐Kun Zhong:** data curation, formal analysis. **Xi‐Dong Wu:** data curation, formal analysis, software, conceptualization, methodology, validation, funding acquisition, resources, visualization. **Tao Liu:** conceptualization, methodology, validation, supervision, funding acquisition, project administration, resources.

## Funding

This study was supported by grants from the National Natural Science Foundation of China (NSFC, 82201248), NSFC Incubation Project of Guangdong Provincial People's Hospital (KY0120220032), and Ganjiang Innovation Talent Program (20241219).

## Ethics Statement

All animal procedures adhered to the guidelines set by IACUC‐Department of Drug Safety Evaluation, Jiangxi Testing Center of Medical Instruments, with the ethics approval number JXYX‐008‐6.3‐12. The animals were housed in the Animal Lab of Nanchang Key Laboratory of Medical Devices Quality Control and Evaluation, China. All animal experiments were conducted in accordance with the guidelines provided by the National Research Council's Guide for the Care and Use of Laboratory Animals.

## Conflicts of Interest

The authors declare no conflicts of interest.

## Data Availability

The data that support the findings of this study are available from the corresponding author upon reasonable request.
